# 

**Published:** 1894-10-20

**Authors:** 


					?ULAOUUr
LMdl
No. 421.^ Vol. XTII. WITH EXTRA NURSING SUPPLEMENT. Satubdat,
Ocr. 20m, 189 4.

				

